# Cystic fibrosis transmembrane conductance regulator (CFTR): Making an ion channel out of an active transporter structure

**DOI:** 10.1080/19336950.2018.1502585

**Published:** 2018-08-28

**Authors:** Paul Linsdell

**Affiliations:** Department of Physiology & Biophysics, Dalhousie University, Halifax, Canada

**Keywords:** ABC protein, CFTR, channel pore, chloride channel, cystic fibrosis, ion channel

## Abstract

Cystic fibrosis is caused by mutations in the cystic fibrosis transmembrane conductance regulator (CFTR). CFTR is a member of the ATP-binding cassette (ABC) family of membrane transport proteins, most members of which function as ATP-dependent pumps. CFTR is unique among human ABC proteins in functioning not as a pump, but as an ion channel. Recent structural data has indicated that CFTR shares broadly similar overall architecture and ATP-dependent conformational changes as other ABC proteins. Functional investigations suggest that CFTR has a unique open portal connecting the cytoplasm to the transmembrane channel pore, that allows for a continuous pathway for Cl^−^ ions to cross the membrane in one conformation. This lateral portal may be what allows CFTR to function as an ion channel rather than as a pump, suggesting a plausible mechanism by which channel function may have evolved in CFTR.

## Introduction

Cystic fibrosis (CF) is a classical autosomal recessive genetic disease, caused by loss-of-function mutations in a single gene, that which encodes the cystic fibrosis transmembrane conductance regulator (CFTR) [1]. CFTR is a member of a large family of membrane transport proteins, the ATP-binding cassette (ABC) family, which is comprised of 48 members in humans subdivided into seven subfamilies (ABCA – ABCG) [2]. Most ABC proteins function as ATP-dependent active transporters, which couple ATP binding and hydrolysis to unidirectional transport of substrates across the membrane [3,4]. Among human ABC proteins, CFTR is thought to be unique in that it has no active transport function, but instead acts as a phosphorylation-regulated, ATP-gated anion channel [5]. CFTR is inactive until phosphorylation of a unique cytoplasmic regulatory region (the R domain), after which it is opened by binding of cytoplasmic ATP and closes following ATP hydrolysis and the release of hydrolysis products [6]. The physiological role of CFTR in epithelial cell salt and water transport [7], the mechanisms and consequences of CFTR dysfunction in CF [8], and pharmacological approaches targeted to CFTR in order to treat CF [9] have been reviewed recently and will not be discussed in this article.

**Figure 1.**
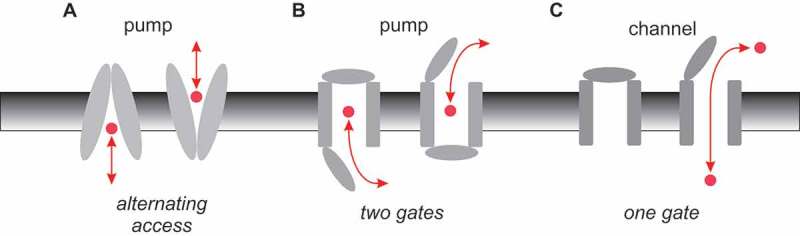
Basic mechanisms of pump and channel function. (a) Alternating access model of pump function. A conformational change in the membrane-spanning parts of the protein (grey) results in substrate (red) being accessible to the intracellular side of the membrane in one conformation (inward-facing), and to the extracellular side of the membrane in the other conformation (outward-facing). (b) This same mechanism can be expressed in terms of outer and inner gates that are never open at the same time (outer gate closed in the inward-facing state, inner gate closed in the outward-facing state). (c) Ion channels can operate with a single gate, which is closed in the channel closed state and open in the channel open state, allowing a continuous aqueous pathway for ion electrodiffusion across the membrane.

Classical membrane biology tells us that active transporters (pumps) work by very different mechanisms than ion channels. Fundamentally, the difference comes down to the way in which access to the substrate translocation pathway is regulated (or gated) [10]. Pumps are most often considered to function by an “alternating access” mechanism, by which a conformational change allows the substrate to be accessible to one side of the membrane or the other (Figure 1(a)). This alternating access mechanism can also be formulated in terms of two classes of gates controlling access to the pathway, which are coupled to each other such that both are never open simultaneously (Figure 1(b)). Ion channels, on the other hand, exist in “closed” and “open” conformational states, that could reflect the opening and closing of a single gate (Figure 1(c)). In the open state, a continuous aqueous pathway connects the bulk solution on the two sides of the membrane – a situation that is incongruous with active transport, and so is presumed never to occur in pumps.

Recent breakthroughs in understanding the atomic structure of CFTR [11–13] indicate that it shares overall structural features with other, active transporter ABC proteins, supporting the widely held view that CFTR likely evolved from a pump ancestor into its current, ion channel form [10,14]. This new structural information, combined with recent functional investigations, has also shed new light on how CFTR functions as an ion channel. In this review, I will summarize these findings and speculate on how CFTR may have jumped the functional divide between pump and channel.

## ABC proteins as classical active transporters

**Figure 2.**
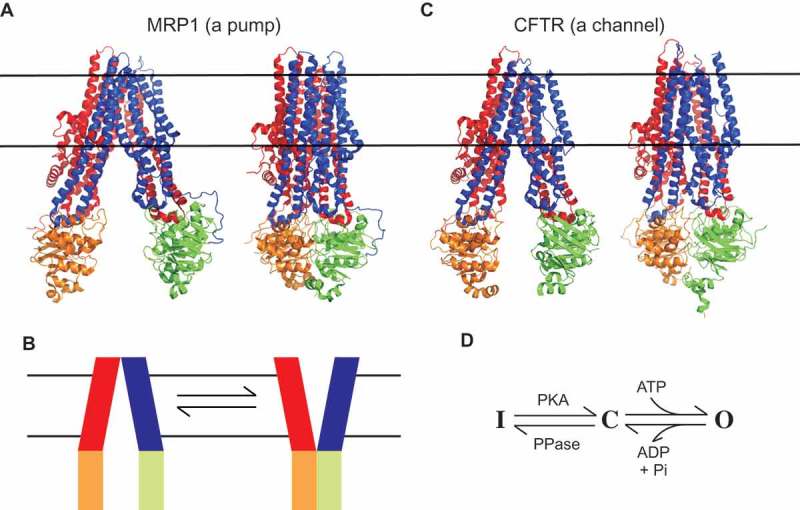
Atomic structures of MRP1 and CFTR. (a) Cryo-EM structures of bovine MRP1 in the inward-facing state (NBDs separated; left) [18] and in the outward-facing state (NBDs dimerized; right) [19]. MSD1, red; MSD2, blue; NBD1, orange; NBD2, green. The approximate location of the cell membrane is indicated by horizontal black lines. To facilitate comparison with the structure of CFTR, an additional N-terminal membrane-associated domain found in MRP1 (MSD0) has been removed from these images. (b) These structures are consistent with an NBD-controlled, alternating access mechanism of active transport by MRP1 [19], as proposed for other ABC proteins [4,15,16]. (c) Cryo-EM structures of human CFTR in an inactive, dephosphorylated state (left) [12], and zebrafish CFTR in a “near-open” state that is closed only at the extracellular ends of the MSDs (right) [13]. The MSDs and NBDs are shown in the same colour scheme as in (a). The cytoplasmic R domain, which is mostly unstructured, is not shown in these images. (d) Basic minimal model for CFTR functional regulation by phosphorylation and by ATP. The channel exists in an inactive state (i) until phosphorylation of the R domain by protein kinase A (PKA). Phosphorylated CFTR transitions to the open burst state (o) following ATP binding, and returns to the closed interburst state (c) following ATP hydrolysis and the release of hydrolysis products (ADP and Pi). ATP-dependent gating continues until the R domain is dephosphorylated by phosphoprotein phosphatases (PPase). This minimal model is a gross oversimplification of current understanding of CFTR channel gating [6,24] to emphasize that the structure shown in (b) (left) corresponds to state **I**, while that on the right is expected to be closest to state **O** [13]. The structure of the phosphorylated, interburst closed state **C**, as described in the text, is not currently known.

Different ABC proteins transport a highly diverse range of substrates, including nutrients, peptides, metabolites, and xenobiotics across cell membranes. They are thought to do this by classical ATP-dependent active transport mechanisms, in which binding and hydrolysis of cytoplasmic ATP control conformational changes in the membrane-spanning parts of the protein to generate alternating access of bound substrate molecules to either side of the membrane [4,15,16]. A relevant example for our purposes is the multidrug resistance protein MRP1 (ABCC1), which is a member of the same ABC subfamily as CFTR (ABCC7) and which acts as an ATP-dependent exporter of a broad range of xenobiotic and chemotherapeutic substances [17]. As with all ABC proteins (including CFTR) [15], MRP1 is made up of two cytoplasmic nucleotide binding domains (NBDs) that interact with ATP, and two membrane-spanning domains (MSDs) that form the substrate translocation pathway (Figure 2(a)). In the ATP-unbound state, the NBDs are separated and the MSDs are in an “inward facing” conformation that allows access of cytoplasmic substrate [18] (Figure 2(a)). ATP binding then causes NBD dimerization and a switch to an “outward facing” conformation of the MSDs [19], allowing substrate to be released into the extracellular solution (Figure 2(a)). This ATP-driven conformational change is therefore fully consistent with an alternating-access mechanism of active transport (Figure. 1(a,b)). The structures of other ABC proteins are also consistent with similar ATP-dependent alternating-access active transport mechanisms [4,15,16].

## The structure of CFTR suggests conformational transitions typical of an ABC protein

Recent atomic-resolution electron cryomicroscopy (cryo-EM) structures of CFTR under different conditions [11–13] are consistent with broadly similar ATP-dependent conformational rearrangements of the NBDs and MSDs (Figure 2(c)). However, CFTR being an ion channel, these rearrangements are presumably associated somehow with channel opening and closing (Figure 2(d)) rather than with active substrate translocation. The first CFTR structures were of dephosphorylated, presumably inactive channels with separated NBDs and inward facing MSDs [11,12] (Figure 2(c)). Following stimulation – and with an NBD2 mutation that interferes with ATP hydrolysis and promotes prolonged channel openings – the channel is observed with dimerized NBDs and with MSDs that are sealed at their intracellular ends where they make contact with the NBDs [13] (Figure 2(c)). This structure is therefore expected more closely to resemble a channel open state, although in fact the channel pore remains sealed close to the extracellular end of the MSDs [13]. For the present discussion, I will refer to this structure as a “near-open” state, as it is thought that only a minor structural rearrangement of this structure is required to open the channel pore [13]. Functionally speaking there is a third state, phosphorylated but closed, that separates “bursts” of openings (state **C** in Figure 2(d)). The structure of this interburst closed state is not currently known. Some functional evidence supports full dissociation of the NBDs following dissociation of the products of ATP hydrolysis and channel closure [20], which might suggest an interburst closed channel structure similar to the inactive structure shown in Figure 2(c) (left). On the other hand, other evidence suggests only partial separation of the NBDs on channel closure [21,22], perhaps suggesting an interburst closed channel structure more similar to the near-open state shown in Figure 2(c) (right) or intermediate between the two structures shown in Figure 2(c). As a result, the exact correspondence of the two CFTR structures shown in Figure 2(c) with these three different overall functional states (**I**nactive, **C**losed, **O**pen; Figure 2(d)) is not completely clear.

## The architecture of the pore supports a “broken pump” mechanism

**Figure 3.**
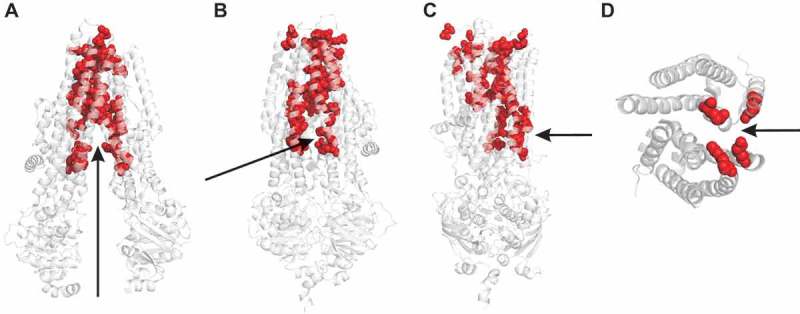
CFTR channel pore-lining residues and location of the cytoplasmic portal. (A-C) Location of putative channel pore-lining amino acid side-chains (red) [23,24] within the CFTR structures shown in Figure 2. (a) In the inactive, dephosphorylated state, a wide central pathway connects the MSDs to the cytoplasm (arrow). (b,c) In the near-open state, this central pathway is closed to the cytoplasm by dimerization of the NBDs and closing of the inner ends of the MSDs. Instead, cytoplasmic access to the pore is via a lateral portal between TMEs 4 and 6. This portal is facing the viewer in (b), and on the right hand side of the MSDs in (c), indicating cytoplasmic access as indicated by the arrows. (d) A cross-section through the TMEs indicates the location of the portal (arrow), as well as important positively charged amino acid side chains (red; K190 (TME3), R248 (TME4), R303 (TME5) and K370 (TME6) in human CFTR) that have been shown to play functional roles in the electrostatic attraction of cytoplasmic anions to the pore [29,30].

Those amino acid residues contributing to the channel pore have been identified by longstanding functional investigation (reviewed in [23,24]). Mapping these residues onto the structure of either the inactive state (Figure 3(a)) or the near-open state (Figure 3(b,c)) suggests a fairly central ion translocation pathway across the membrane lined by several transmembrane α-helices (TMs). Because both of these structures are closed near the extracellular end of the TMs, this outer region is the likely location of the channel gate, consistent with functional investigations [25,26].

Since the inactive state exhibits inwardly facing MSDs, the channel pore appears wide open to the cytoplasm in this state (Figure 2(c), 3(a)). However, the unphosphorylated R domain (which is unstructured in the cryo-EM structure) [12] may plug this apparent pathway and prevent cytoplasmic access to the pore. Since in the near-open state the inner ends of the MSDs come into close contact with each other – and also because the NBD dimer interacts with these tightly associated cytoplasmic extensions of the MSDs – this apparent central pathway from the cytoplasm to the pore is closed off (Figure 2(c), 3(b,c)). How, then, do cytoplasmic anions access the open channel pore? Based on atomic homology modeling and molecular dynamics studies, it was proposed that such access may be provided not by a central pathway, but instead by one or more (possibly up to four) lateral entrances between individual transmembrane domain cytoplasmic extensions (TMEs) [27,28]. The first functional evidence for the existence of such cytoplasmic lateral portals to the pore came from substituted cysteine accessibility mutagenesis work that identified pore-lining, positively charged amino acid side chains within the TMEs and apparently surrounding two potential portals [29]. Subsequent, more detailed biophysical analysis suggested that there is only one functional portal involved in the entry of cytoplasmic Cl^−^ ions into the pore, and indicated that four positively charged residues – K190 (TME3), R248 (TME4), R303 (TME5) and K370 (TME6) – play major roles in attracting cytoplasmic Cl^−^ ions to the pore via this portal [30]. The cryo-EM structure of the near-open state confirms the presence of a single large portal between TME4 and TME6 [13], apparently lined by these four functionally important positively charged residues (Figure 3(d)). No similarly sized portal was observed on the “opposite” side of the TMEs (ie. between TME10 and TME12) [13], again consistent with functional results suggesting a single functionally relevant portal [30]. Once within the interior of the protein, Cl^−^ ions appear to follow a fairly central route through the TMEs towards the cell membrane and the TM-lined central pore [31].

Lateral portals or fenestrations have been identified connecting other ion channel pores to the cytoplasm (eg [32–36].), however the portal identified in Figure 3(d) appears unique among ABC proteins and prompts speculation concerning the channel function of CFTR. From cursory inspection of the structures of MRP1 and CFTR shown in Figure 2, it is impossible to guess which is a pump and which a channel, which suggests that CFTR has retained major aspects of an ATP-dependent rearrangement of the MSDs from its ABC ancestry. However, this this rearrangement of the MSDs must open a channel pore in CFTR, rather than switching the accessibility of a substrate binding site within the MSDs as in MRP1 and other ABC proteins (Figure 2(b)). The inward facing state of CFTR is sealed at the outer end of the MSDs (Figure 2(c), 3(a)) (as it is in other ABC proteins (Figure 2(a))), consistent with a closed outer gate. ATP-dependent NBD dimerization brings together the inner ends of the MSDs (Figure 2), which in other ABC proteins generates an outward facing state with a closed inner gate (Figure 2(a,b)). However, in CFTR the presence of the portal connecting the pore with the cytoplasm in the pseudo-inward facing state (Figure 3(b-d)) effectively short-circuits the inner gate, by allowing for a continuous aqueous pathway between the cytoplasm and the extracellular solution. This description is a slightly new twist on the longstanding “broken pump” hypothesis, which postulates that CFTR evolved from a pump into a channel when one of the two gates regulating alternating access to the pore (Figure 1(b)) became dysfunctional [10,14,37]. If the two gates in question are considered to be located at the outer and inner ends of the MSDs respectively (Figure 2), then neither of these gates may have become “broken” – both still open and close in response to ATP action at the NBDs. Instead, the inner gate in CFTR is now irrelevant in terms of regulating access to the transport pathway, since the portal provides a “leak” pathway between this gate and the transmembrane pore. Possibly CFTR should be considered a “leaky pump” rather than a “broken pump” (although there is no evidence that it is still able to pump anything).

**Figure 4.**
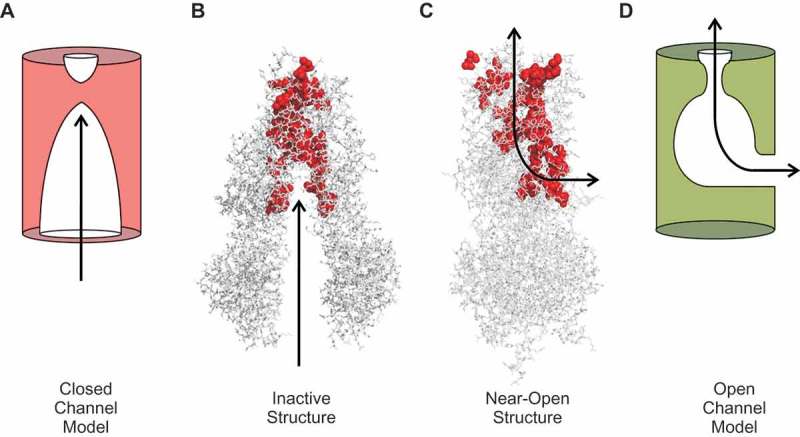
Proposed mechanisms of CFTR channel opening and closing. (a) Cartoon functional model of the pore in the channel closed state. The pore is wide open to the cytoplasm, but closed close to its extracellular end. (b) This cartoon model is somewhat reminiscent of the inward-facing structure of inactive CFTR (putative pore-lining amino acid side chains shown in red). (c) In the near-open structure, cytoplasmic access to the pore is via a lateral portal. (d) Cartoon functional model of the open channel pore incorporating a single lateral portal on the cytoplasmic side, a relatively wide inner vestibule, and narrow selectivity filter region close to the extracellular end of the pore. See [23] for more details concerning functional models of the open channel pore.

Speculatively, it could be proposed that the evolution from an active transporter into an ion channel was associated with the appearance of the portal that allowed a continuous pathway for small substances to exist in one conformation (Figure 4). This pathway could have been fine-tuned into an anion selective channel pore, for example by positioning of positively charged amino acid residues at key positions, and of an appropriately size-selective barrier or “selectivity filter” [23]. Evolution of the R domain – which is also unique to CFTR – may have allowed for an additional level of regulation, by substances that impinge on the cyclic AMP second messenger system [38].

## Conclusions and future perspectives

Classically considered very different types of membrane transport proteins, the distinction between pumps and channels has become somewhat blurred over recent times [10,39–44]. Advances in understanding both the structure and the function of the Cl^−^ permeation pathway in CFTR has suggested one way in which a membrane transport protein may have been able to make the transition between the two worlds of pump and channel. In the near-open state, CFTR has all the hallmarks of a “normal” ion channel, such as a relatively narrow selectivity filter, a localized gate, and charged residues that electrostatically attract permeating anions to the pore (Figure 4(d)). From its active transporter ancestry, it appears to have retained a coupling between ATP binding and hydrolysis and conformational changes in the substrate translocation pathway, and large-scale rearrangements of the MSDs that are reminiscent of the classical alternating access mechanism of a pump (Figure 2).

Because ion channels are routinely studied using electrophysiological approaches, and because ionic current flows only through the open state, functional models of the open CFTR channel are relatively well developed [23] (Figure 4(d)). How the structure of the channel pore changes when the channel closes is less clear. Both structural [13,28] and functional [25,26] data suggests that the gate that shuts off the pore to permeating Cl^−^ ions is located close to the extracellular end of the MSDs. It is also known that large cytoplasmic substances can penetrate deep into the pore in both the open state and the closed state [45], suggesting that the inner pore remains wide open to the cytoplasm throughout the gating cycle. These findings are consistent with a model of the closed channel pore (Figure 4(a)) that resembles the inactive, NBDs-separated structure of CFTR (Figure 4(b)). However, it is not yet clear if this structure truly reflects the closed-but-phosphorylated, interburst closed state of CFTR (Figure 2(d)). For example, some evidence suggests [21,22] that the NBDs do not become completely separated during interburst closures (as they appear to be in the inactive, dephosphorylated structure (Figure 2-4), as well as in MRP1 in the ATP-unbound state (Figure 2(a))). Thus, the closed state structure may be somehow intermediate between the two structures shown in Figure 2(c). As a result, it is not known if cytoplasmic substances can access the closed channel pore via a central pathway (as shown in Figure 4(a,b)), or via the same portal that they are obligated to use to access the open channel pore (Figure 4(c,d)). Additional structures of CFTR – most importantly, of the interburst closed state structure – will be most useful in resolving these outstanding questions. Additional structural information may also shed new light on the mechanism of operation of the channel gate (which still appears closed in the near-open state of Figure 2-4), as well as the role of the R domain (which is absent from all CFTR structures presented in this article). Identification of key structural differences between the open and closed channel states may also help in the development of CFTR “potentiators” – substances that bind to CFTR and increase its activity that are currently used for the treatment of CF patients [9,46] – in particular if such substances act by binding preferentially to the open channel and stabilizing its structure [45].
